# Postpartum aortic dissection in a patient without Marfan’s syndrome

**DOI:** 10.4274/tjod.35336

**Published:** 2016-12-15

**Authors:** Mihriban Yalçın, Melih Ürkmez, Kaptanı Derya Tayfur, Serkan Yazman

**Affiliations:** 1 Ordu State Hospital, Clinic of Cardiovascular Surgery, Ordu, Turkey

**Keywords:** Aortic dissection, Marfan’s syndrome, postpartum

## Abstract

Aortic dissection can occur in pregnancy or during the postpartum period without pre-existing disease and it is a rare but potentially life-threatening event. Herein, we present a young woman without Marfan’s syndrome who developed a postpartum ascending aortic dissection 5 days after cesarean section.

## INTRODUCTION

Aortic dissection is the separation of the aortic wall layers and formation of a true lumen and a false lumen. Acute aortic dissection in pregnant women is a rare but potentially life-threatening event. It is usually related to severe hypertension due to preeclampsia, coarctation of the aorta or connective tissue disorders such as Marfan’s syndrome. Aortic dissection may occur at any time during gestation (5% in the first trimester, 10% in the second trimester, 50% in the third trimester, and 20% postpartum)^(1)^. Postpartum aortic dissection occurs between day 1 and day 42 after either vaginal or cesarean section delivery. We report a young woman without Marfan’s syndrome who developed postpartum ascending aortic dissection 5 days after she delivered a healthy female infant by cesarean section.

## CASE REPORT

**Figure 1:**
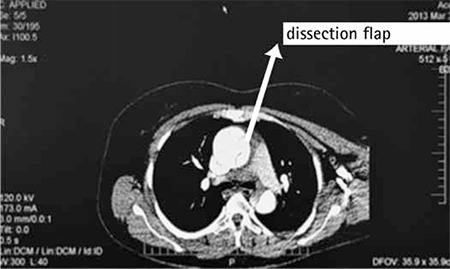
The computed tomography angiography showing the dissection

A multiparous women aged 40 years who had an uneventful cesarean section 5 days previously was admitted to the emergency department with severe chest and back pain. According to information gained from the patient, she had regular antenatal checkups, she had no history of serious illness (chronic hypertension, cardiac disease, kidney disease, or connective tissue disorder), operations, or hospitalization. She had no family history of connective tissue diseases or signs of Marfan’s syndrome, and did not have preeclampsia or perinatal or prior heart disease. When she arrived at the hospital, her blood pressure was 160/60 mmHg, heart beat was 65 beats per minute, electrocardiography was normal, and D-dimer was high (2.860 ng/mL). The patient was referred to our hospital with a diagnosis of pulmonary embolism. Thoracal computed tomography angiography (CTA) showed type A aortic dissection (Figure 1). There was a dissection flap that started from the ascending aorta and extended into the iliac arteries. The diameter of ascending aorta was 43 mm. Transthoracic echocardiography revealed that her ejection fraction was 65%, and there was minimal regurgitation of the aortic valve. She was taken to the operating room.

Under deep hypothermic circulatory arrest, an ascending aortic replacement was performed. Four hours postoperatively, she was taken into surgery again due to major bleeding and hypotension. Surgery revealed that the dissection had progressed to the aortic root. Cardiopulmonary bypass was begun, and a Bentall operation was performed. Unfortunately, the patient could not tolerate this second operation and died of uncontrollable bleeding.

## DISCUSSION

Aortic dissection can occur in pregnancy or during the postpartum period without pre-existing disease due to hormonal changes, regardless of whether delivery was vaginal or by cesarean section. After hypertension and Marfan’s syndrome, pregnancy is the most common risk associated with aortic dissection, a potentially deadly event.

The increase of estrogen and progestogen in the third trimester of pregnancy may add additional risk because the aorta expresses oestrogen and progestogen receptors^(2)^. Peripartum hormonal changes can cause fragmentation of reticular fibers, decreases in the amount of acid mucopolysaccharides, and damage to the normal shapes of elastic fibers, thereby increasing the risk of aortic dissection. These changes in the structure of the aorta during pregnancy have been reported to be similar to the medial degeneration pattern found in patients with aortic dissection.

Cardiovascular stresses such as pressure, heart rate, stroke volume, cardiac output, left ventricular mass, and blood volume are increased by pregnancy, and may cause hemodynamic stress on the aortic wall^(2)^. With the termination of puerperal uteroplacental circulation and uterine contraction, along with interstitial fluid absorption, the circulating volume could be increased by 15-25% within 72 h of delivery in a postpartum woman^(3)^. Thus, the third trimester of pregnancy or immediate postpartum stage is the most common interval during which aortic dissection occurs.

During pregnancy, the aorta and the vessel wall structures are generally weaker and more sensitive to hemodynamic forces. These pregnancy-related hemodynamic stresses and hormonal changes are the main factors for the development of aortic dissection^(4)^. CTA and transesophageal echocardiogram are the gold standard for the diagnosis of aortic dissection. CTA provides important information about the extent of dissection, the relation between the true and false lumen, and aortic branch compromise.

The complications of aortic dissection are aortic rupture, aortic regurgitation, acute myocardial infarction, tamponade, and end-organ ischemia. Back pain, chest pain, lower extremity ischemia and paraplegia are common symptoms of aortic dissection. If dissection involves the great vessels to the brain, loss of consciousness or signs of stroke may be seen. Survival is directly related to the timing of emergency intervention because the mortality rate increases 1 to 2% every hour during the first 24 to 48 hours after dissection^(5)^. Open surgical repair of type A dissections is recommended.

Yuan^(3)^ evaluated 27 patients with postpartum aortic dissection. Pain was the most common symptom at onset, as it was in our petient. Sixteen (59.3%) patients had type A aortic dissections and four (14.8%) died^(3)^.

Yang et al.^(6)^ reported 11 paients who had aortic dissection during the course of pregnancy or puerperium. They found 6 patients during the postpartum stage, 4 of whom had type A aortic dissections and 2 died. Immer et al.^(7)^ reported 5 patients with postpartum type A aortic dissections, one patient died.

Late presentation, delayed or misdiagnosis may be associated with postpartum aortic dissection because of its rarity^(8)^. The differential diagnoses of severe chest pain include acute myocardial infarction, pulmonary embolism, and aortic dissection^(8)^. Normal electrocardiogram cardiac enzymes are needed to exclude myocardial infarction. Normal coagulation test results and D-dimer level help rule out pulmonary embolism; the level of D-dimer was high in our case.

In the present case, the cause of death was the progress of dissection and uncontrolled bleeding. The patient reported here was known to have had normal blood pressure throughout her pregnancy and had no risk factors such as trauma, smoking, drug or alcohol abuse. She also had no family history of Marfan’s syndrome.

## CONCLUSION

This case suggests that acute aortic dissection can occur postpartum in young women who are normotensive and without Marfan’s syndrome. Therefore, it is important to consider aortic dissection as a possible diagnosis during pregnancy and also after delivery. Aortic dissection is easily misdiagnosed as other cardiac, muscular, neurologic, esophageal or renal diseases because it may present with different clinical symptoms, such as back, chest, epigastric and abdominal pain, and cardiac arrest, which could potentially lead to the death of a new mother. Thus, obstetricians should consider aortic dissection as a possible diagnosis when these symptoms present in a postpartum woman.

We acknowledge the medical writing assistance provided by American Manuscript Editors (www.americanmanuscripteditors.com) for the final draft of the manuscript. The authors report no declarations of interest.

## References

[1] Wanga S, Silversides C, Dore A, Waard V, Mulder B (2016). Pregnancy and Thoracic Aortic Disease: Managing the Risks. Can J Cardiol.

[2] van Hagen IM, Roos-Hesselink JW (2014). Aorta pathology and pregnancy. Best Pract Res Clin Obstet Gynaecol.

[3] Yuan SM (2013). Postpartum aortic dissection. Taiwan J Obstet Gynecol.

[4] Gelpi G, Pettinari M, Lemma M, Mangini A, Vanelli P, Antona C (2008). Should pregnancy be considered a risk factor for aortic dissection? Two cases of acute aortic dissection following cesarean section in non-Marfan nor bicuspid aortic valve patients. J Cardiovasc Surg (Torino).

[5] Fuster V, Andrews P (1999). Medical treatment of the aorta. I. Cardiol Clin.

[6] Yang G, Peng W, Zhao Q, Peng J, Xiang X, Chai X (2015). Aortic dissection in women during the course of pregnancy or puerperium: a report of 11 cases in central south China. Int J Clin Exp Med.

[7] Immer FF, Bansi AG, Immer-Bansi AS, McDougall J, Zehr KJ, Schaff HV, et al (2003). Aortic dissection in pregnancy: analysis of risk factors and outcome. Ann Thorac Surg.

[8] Kang BH, Lee MA, Rhee YE, Noh HT (2011). Unexpected acute aortic dissection after elective cesarean section delivery: report of a case and review of the literature. Korean J Obstet Gynecol.

